# Pre-Treatment Prediction of Breast Cancer Response to Neoadjuvant Chemotherapy Using Intratumoral and Peritumoral Radiomics from T2-Weighted and Contrast-Enhanced T1-Weighted MRI

**DOI:** 10.3390/cancers17091520

**Published:** 2025-04-30

**Authors:** Deok Hyun Jang, Christopher Kolios, Laurentius O. Osapoetra, Lakshmanan Sannachi, Belinda Curpen, Ana Pejović-Milić, Gregory J. Czarnota

**Affiliations:** 1 Physical Sciences, Sunnybrook Research Institute, Toronto, ON M4N 3M5, Canada; 2 Department of Radiation Oncology, Sunnybrook Health Sciences Centre, Toronto, ON M4N 3M5, Canada; 3 Department of Physics, Toronto Metropolitan University, Toronto, ON M5B 2K3, Canada; 4 Department of Medical Biophysics, Faculty of Medicine, University of Toronto, Toronto, ON M5T 1P5, Canada; 5 Department of Medical Imaging, Sunnybrook Health Sciences Centre, Toronto, ON M4N 3M5, Canada; 6 Department of Radiation Oncology, Faculty of Medicine, University of Toronto, Toronto, ON M5T 1P5, Canada

**Keywords:** radiomics, MRI, breast cancer, neoadjuvant chemotherapy, response prediction, machine learning

## Abstract

(1) Background: Neoadjuvant chemotherapy (NAC) is an integral part of breast cancer management, and response to NAC is an important prognostic factor associated with improved survival outcomes. However, the current standard for response assessment relies on post-surgical histopathological analysis, which limits early therapeutic decision-making and treatment personalization. This study aimed to develop and evaluate a machine learning model that integrates pre-treatment MRI radiomics and clinical features to predict response to NAC in breast cancer patients. (2) Methods: In this study, a machine learning model was developed to predict breast cancer response to NAC using pre-treatment magnetic resonance imaging (MRI) radiomics and clinical data. Radiomic features were extracted from contrast-enhanced T1-weighted (CE-T1) and T2-weighted (T2) MRI sequences using both intratumoral and peritumoral segmentations. Furthermore, this study uniquely examined two response assessment criteria: (1) pathologic complete response (pCR) versus non-pCR, and (2) clinical response versus non-response. A total of 254 patients with biopsy-confirmed breast cancer who completed NAC were included. Radiomic features (*n* = 400) and clinical features (*n* = 7) were analyzed to build a predictive model employing the XGBoost classifier. Performance was measured in terms of accuracy, precision, sensitivity, specificity, F1-score, and AUC. (3) Results: The integration of radiomic features with clinical data significantly enhanced the predictive performance. For pCR and non-pCR prediction, the combined features model achieved an accuracy of 80% and AUC of 0.85, outperforming both the clinical features model (Accuracy = 68%, AUC = 0.81) and radiomic features model (Accuracy = 66%, AUC = 0.60). Similarly, for the clinical response and non-response prediction, the combined features model achieved an Accuracy of 74% and AUC of 0.75, outperforming both the clinical features model (Accuracy = 63%, AUC = 0.68) and radiomic features model (Accuracy = 66%, AUC = 0.57). (4) Conclusions: These findings highlight the synergistic effect of integrating clinical data and MRI-based radiomics to improve pre-treatment NAC response prediction, which has the potential to enable more precise and personalized treatment strategies.

## Simple Summary

Response to NAC is a crucial predictor of survival outcomes. Currently, the standard response assessment method relies on post-surgical histopathology, which limits early treatment modifications. This study developed a machine learning model to predict NAC response using pre-treatment MRI radiomics and clinical data. Radiomic features from T1-weighted and T2-weighted MRI were analyzed alongside clinical variables in 254 breast cancer patients. Two response criteria were examined: pathologic complete response (pCR) and clinical response. The combined model, integrating radiomics and clinical data, outperformed individual models, achieving an AUC of 0.85 for pCR prediction and 0.75 for clinical response prediction. These findings highlight the potential of radiomics-based response prediction models for enabling precise, personalized NAC strategies.

## 1. Introduction

Breast cancer is the most commonly diagnosed cancer and the leading cause of cancer-related death in women worldwide [1]. The Global Cancer Statistics from the International Agency for Research on Cancer (IARC) estimated 2.3 million new breast cancer diagnoses and 666,000 breast cancer-related deaths in 2022 [2]. Despite increasing incidence, mortality rates have declined, particularly in developed countries. This decline is largely attributed to the implementation of mammographic screening programs, which facilitate early detection, as well as significant advancements in treatment options [2,3]. The current standard treatment for nonmetastatic breast cancer typically involves a combination of chemotherapy, endocrine therapy, targeted therapy, surgery, and radiation therapy, depending on the stage and subtype [4]. Neoadjuvant chemotherapy (NAC), which is defined as chemotherapy administered prior to surgery, is primarily used for patients with locally advanced or inflammatory breast cancer and is also considered in selected early-stage cases with high-risk tumor biology, such as human epidermal growth factor receptor 2 (HER2) enriched or triple-negative subtypes [5]. This systemic therapy complements surgery, which remains the central curative approach in breast cancer management, by reducing tumor size, downstaging the disease, and potentially enabling breast-conserving surgery or improving overall operability [6]. In recent years, NAC has advanced through the incorporation of targeted therapies and immunotherapy, which have the potential to improve long-term outcomes, particularly for aggressive breast cancer subtypes.

Beyond its role in tumor reduction, NAC provides valuable prognostic insights based on tumor response to systemic therapy [7]. NAC response is a strong predictor of survival, particularly for patients achieving pathologic complete response (pCR), which is defined as the complete eradication of invasive disease and lymph node involvement [8,9]. Patients who achieve pCR demonstrate improved event-free survival (EFS) and overall survival (OS), particularly those with HER2-enriched or triple-negative subtypes [10]. However, only a small percentage of patients achieve pCR following NAC [9,10], and the majority achieve partial response, defined as a range of responses between pCR and stable disease [11,12]. Partial response is associated with better survival than stable or progressive disease but worse outcomes than pCR [11,12]. Since NAC response is strongly associated with survival outcomes, early identification of non-responsive patients may allow adaptive therapy that has the potential to enhance response and ultimately improve survival outcomes. A range of diagnostic tools, including MRI [13] and emerging approaches such as liquid biopsy [14], has the potential to provide minimally invasive assessments of response and offer insight into treatment efficacy. However, post-surgical histopathological examination, which definitively confirms the absence or presence of residual invasive disease remains the clinical gold standard for assessing tumor response to NAC. Therefore, early prediction of tumor response to NAC before therapy initiation has attracted significant clinical interest.

Radiomics is an emerging field that involves the extraction of quantitative features from radiologic images, which captures underlying pathophysiological characteristics that are not apparent to human perception [15,16]. Advances in computational power and machine learning techniques have propelled the growth of the field, leading to its investigation into various diseases [17]. In breast cancer, radiomics has been explored for applications such as disease detection and characterization, response prediction, and prognostication [18,19,20,21]. Furthermore, breast cancer response prediction has been investigated using various imaging techniques including quantitative ultrasound (QUS) [22,23], computed tomography (CT) [24], and magnetic resonance imaging (MRI) [25,26,27]. Among these, MRI is a leading modality for breast cancer radiomics due to its superior soft tissue contrast [28] and routine use in standard breast cancer management.

This study aims to develop a robust NAC response prediction model based on pre-treatment MRI radiomics and clinical information. A multi-sequential and multi-segmental approach was employed, extracting radiomic features from contrast-enhanced T1-weighted (CE-T1) and T2-weighted (T2) MRI sequences using both intratumoral and peritumoral segmentations. Unlike many prior MRI radiomics studies on breast cancer that focus solely on pCR prediction, this study uniquely examines both pCR-based and clinical response-based assessment criteria. The first criterion evaluates pathologic complete response versus non-pCR, while the second assesses clinical response versus non-response, where “response” includes both complete and partial responses. The prognostic significance of this second response assessment criterion has been demonstrated in a previous QUS study, which found a statistically significant difference in recurrence-free survival (RFS) between responders and non-responders [22]. Since partial response constitutes the majority of NAC outcomes, integrating both assessment criteria provides a more comprehensive evaluation of treatment response and helps identify non-responders, the group most vulnerable to poor outcomes. Ultimately, radiomics-based NAC response modeling has the potential to support clinical decision-making and further advance precision medicine in breast cancer management. Reliable pre-treatment identification of patients unlikely to benefit from standard therapy enables early therapeutic modification, which may improve response rates and survival outcomes while reducing adverse effects.

## 2. Materials and Methods

### 2.1. Patient Selection and Clinical Features

This was a single-institution study conducted at Sunnybrook Health Sciences Centre, Toronto, Canada. Institutional research ethics board approval was obtained before study initiation. Patients from 2013 to 2019 with biopsy-proven LABC and high-risk early-stage tumors, characterized by features such as HER2 positivity, hormone receptor negativity, or high histologic grade were considered for inclusion in this study. LABC was defined according to AJCC staging as stage IIB to IIIC disease, including tumors with extensive nodal involvement or direct extension to the chest wall or skin but without distant metastasis. Biopsy was performed at the time of diagnostic mammography and ultrasound to confirm malignancy prior to initiation of any treatment. To ensure confidentiality, patient information was anonymized by removing any potential identifiers before conducting the analysis. Eligible patients (i) had both biopsy and MRI performed at the institution before NAC, (ii) completed standard anthracycline and taxane-based NAC, and (iii) underwent surgery at the institution. As per institutional protocol, two regimens were considered standard NAC. The first regimen consisted of three cycles of fluorouracil, epirubicin, and cyclophosphamide (FEC), followed by three cycles of docetaxel, with each cycle administered every three weeks. The second, dose-dense regimen included four cycles of doxorubicin and cyclophosphamide (AC), followed by four cycles of paclitaxel, administered every two weeks. Both regimens were supplemented with trastuzumab for HER2-positive patients as targeted therapy. Patients were excluded from the study for (i) inadequate MRI quality or the presence of artifacts, (ii) missing pre-treatment or post-surgery pathological data, or (iii) the presence of breast enhancement implants. Patients with breast implants were excluded as they may introduce imaging artifacts and distort tumor shape, compromising the reliability of radiomic feature extraction.

Clinical information, which serves as the basis for clinical decision-making throughout breast cancer management, was incorporated into the analysis as clinical features. As part of the standard of care, estrogen receptor (ER), progesterone receptor (PR), and HER2 status were determined from the initial biopsy via immunohistochemistry (IHC) by pathologists. These receptor statuses, which are well-established predictors of tumor response to NAC, were dichotomized as positive or negative in the clinical feature set. Other clinical information, including age, histological grade (G1, G2, and G3), tumor size (in mm), and clinical nodal status (N0, N1, N2, and N3) according to the American Joint Committee on Cancer (AJCC) TNM staging was also incorporated into the clinical feature set. As a result, a total of seven clinical features were used. Clinical data of the patients were retrieved from the institutional electronic patient records.

### 2.2. Response Evaluation

In this study, two criteria for tumor response were evaluated. The first criterion assessed whether patients achieved pCR, which was defined as the absence of invasive cancer in both the breast and axillary nodes. The presence of residual ductal carcinoma in situ (DCIS) after NAC was not considered in this definition of pCR. Patients were then categorized into pCR and non-pCR groups based on pathology, where the non-pCR group included both partial responders and non-responders. This response assessment was designated as “criterion 1”. The second assessment criterion was a modified grading system [29] that compared the radiologically assessed pre-treatment tumor size with the pathologically assessed post-treatment tumor size. This modified system was based on the RECIST criteria [30], which compare radiologically evaluated pre-treatment and post-treatment tumor sizes. The pre-treatment tumor size was determined from the longest dimension measured on pre-treatment MRI, and the post-treatment residual tumor size was retrieved from pathological reports of the surgical specimen. Similar to RECIST criteria, a tumor size reduction of 30% or more was determined as a response, including both pCR and partial response, whereas a reduction less than 30% was classified as a non-response, also encompassing both stable disease (no size change) and progressive disease (increased size). In addition, when residual tumor cellularity was below 1%, it was considered a response [29]. This response evaluation criterion was designated as “criterion 2”.

### 2.3. MRI Acquisition and Analysis

**Figure 1:**
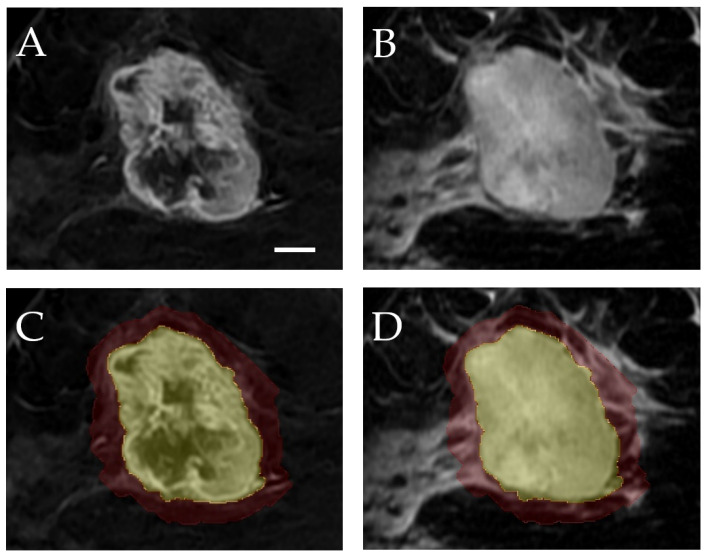
Visualization of tumor segmentation in MR images. (**A**) Contrast-enhanced T1-weighted (CE-T1) image; (**B**) T2-weighted image; (**C**) CE-T1 image with intratumoral (yellow) and peritumoral (red) segmentations; (**D**) T2 image with the same segmentations. A scale bar corresponding to 1 cm is shown in the bottom right corner of image (**A**).

MR data were acquired using either a Signa (GE Healthcare, Chicago, IL, USA) or an Aera (Siemens Healthcare, Erlangen, Germany) 1.5 T scanner, each equipped with a dedicated 8-channel breast coil. T2-weighted (T2) and contrast-enhanced T1-weighted (CE-T1) images were retrieved from the institutional Picture Archiving and Communication System (PACS) for radiomic analysis. Image acquisition parameters for T2 and CE-T1 images are detailed in Supplementary Table S1. The region of interest (ROI), encompassing the entire tumor volume, was manually delineated slice-by-slice based on CE-T1 images. By default, T2 images were co-registered with CE-T1 images, and the same ROI was applied. Tumor delineation was performed using 3D Slicer (version 5.2.1), an open-source software for radiographic image analysis [31]. The ROI was validated by an expert radiologist and oncologist. Additionally, a peritumoral area was generated by an isotropic expansion of the original tumor boundary by 5 mm based on previous work indicating the importance of this region [25,32]. Figure 1 presents example CE-T1 and T2 images with intratumoral and peritumoral segmentations.

### 2.4. Radiomic Feature Extraction

Prior to feature extraction, pre-processing steps including N4 bias field correction, Z-score intensity standardization, and voxel resampling to 1 mm × 1 mm × 1 mm were applied to the T2 and CE-T1 images. Additionally, image intensities were discretized using a bin count of 128. These steps were implemented to ensure the standardization of image quality, particularly to account for variations in image acquisition from the two MRI scanners. Radiomic feature extraction was performed using the PyRadiomics library (version 3.0.1), a Python-based open-source tool [33]. For each patient, three classes of radiomic features were determined per sequence and per region of interest (ROI) in both intratumoral and peritumoral regions; these included 14 shape-based features, 18 first-order features, and 75 second-order features. Shape-based features were used to describe the three-dimensional geometry and size of the ROI [17] and did not rely on the intensity values of the voxels. First-order features provided statistical descriptors of voxel intensity distribution within the ROI [17]. Second-order features, also known as texture features, captured the spatial relationship of voxel intensities within the ROI [17]. This set of 75 second-order features included 24 Gray Level Co-occurrence Matrix (GLCM) features, 16 Gray Level Run Length Matrix (GLRLM) features, 16 Gray Level Size Zone Matrix (GLSZM) features, 14 Gray Level Dependence Matrix (GLDM) features, and 5 Neighboring Gray Tone Difference Matrix (NGTDM) features. In total, 400 radiomic features were obtained from each patient across T2 and CE-T1 sequences and from both intratumoral and peritumoral ROIs (28 shape-based features, 72 first-order features, and 300 second-order features). It should be noted that the shape features were independent of the two MRI sequences, and they were determined solely based on the two segmentations. A complete list of extracted features is provided in Supplementary Table S2.

### 2.5. Machine Learning Classification

In this study, three subsets of features were used to predict pCR versus non-pCR and response versus non-response: the first set with seven clinical features, the second set with 400 radiomic features, and the third set with 407 clinical and radiomic features. Given the well-established importance of the clinical features in response prediction, a subset analysis was conducted to examine the potential additive value of incorporating radiomic features into the prediction model. For labeling purposes, non-pCR and non-response were designated as the positive cases, and pCR and response were designated as the negative cases, reflecting the greater clinical interest in identifying less-responsive patients.

The classification performance of the model was evaluated using 10 partitions, with 80% of the data allocated as a training set and 20% held out as a test set. The data were partitioned with stratification to preserve the ratio of majority and minority classes. Feature selection, hyperparameter tuning, and model building were performed on the training set, and classification performance was evaluated on the hold-out test set. Performance was measured in terms of accuracy, precision, sensitivity, specificity, F1-score, and AUC. These metrics were averaged across the 10 independently trained partitions in order to assess the overall model performance.

After data partitioning, a robust scaler algorithm was applied to standardize the range of feature values in the dataset. Two stages of feature selection were then implemented to reduce the number of features for final model training, thereby reducing the risk of overfitting and improving computational efficiency. The first stage of feature selection reduced the number of features to 25 using the Minimum Redundancy Maximum Relevance (mRMR) algorithm, a filter-based feature selection method using mutual information to assess both feature relevance to the target variable (response) and the redundancy among the features [34]. It ranked features by maximizing relevance while minimizing redundancy between features. The second stage of feature selection employed recursive feature elimination (RFE), a wrapper-based method that identifies the most important features by recursively removing the least important features. RFE was conducted using the Extreme Gradient Boosting (XGBoost) classifier with 5-fold cross-validation within the training set, further reducing the number of features without a pre-specified target number. Feature selection was repeated independently in each partition, and the frequency of selection across 10 iterations was recorded for each feature, allowing assessment of feature stability and identification of consistently selected predictors. Finally, the XGBoost classifier [35], a well-established algorithm known for its high classification performance [36,37] and computational efficiency, was used to train the model based on the selected features. Hyperparameter tuning was performed using grid search with 5-fold cross-validation in the training set. The hyperparameter settings are provided in Supplementary Table S3.

### 2.6. Statistical Analysis

Differences in the distributions of clinical and selected radiomic features were assessed between the pCR and non-pCR groups based on criterion 1 and between the responders and non-responders based on criterion 2. The Shapiro–Wilk test was used to evaluate the normality of feature distributions. To assess significant differences between response and non-response groups, the Pearson χ^2^ test was applied for categorical variables, while independent two-tailed *t*-tests and Mann–Whitney U-tests were used for normally and non-normally distributed continuous features, respectively. Additionally, classification performance across the three feature sets was compared using paired two-tailed t-tests. Statistical significance was defined as *p* < 0.05.

## 3. Results

### 3.1. Clinical Characteristics

**Figure 2:**
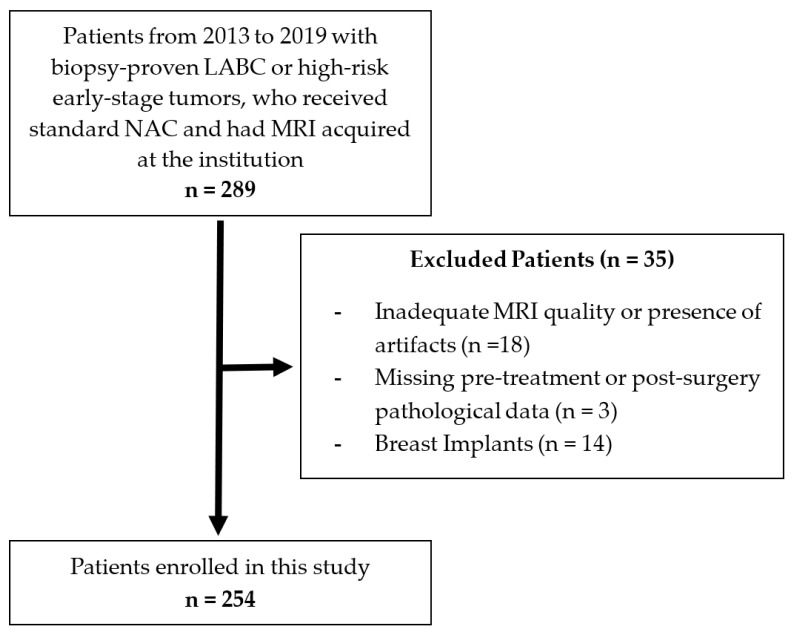
Patient selection flowchart with exclusion criteria.

As shown in Figure 2, a total of 254 patients were enrolled in this study according to the inclusion and exclusion criteria. The clinical characteristics of the cohort are summarized in Table 1 for response assessment criterion 1 (pCR versus non-pCR) and Table 2 for criterion 2 (response versus non-response). According to criterion 1, 63 patients (24.8%) were labeled as pCR, and 191 patients (75.2%) were labeled as non-pCR. According to criterion 2, 183 patients (72.0%) were labeled as responders, and 71 patients (28.0%) were labeled as non-responders. Both criteria exhibited similar proportions for the majority class: 75.2% for non-pCR under criterion 1 and 72.0% for responders under criterion 2.

For criterion 1, a statistically significant difference between the pCR and non-pCR groups was observed in all clinical characteristics except for age. For criterion 2, a statistically significant difference between responders and non-responders was observed in histologic grade, as well as ER, PR, and HER2 receptor status.

### 3.2. Classification Results

**Figure 3:**
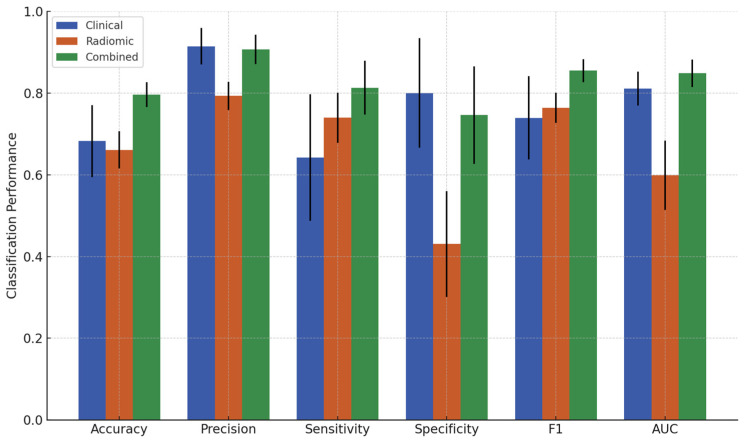
Performance metrics comparison of clinical, radiomic, and combined feature sets for predicting pCR vs. non-pCR (criterion 1).

**Figure 4:**
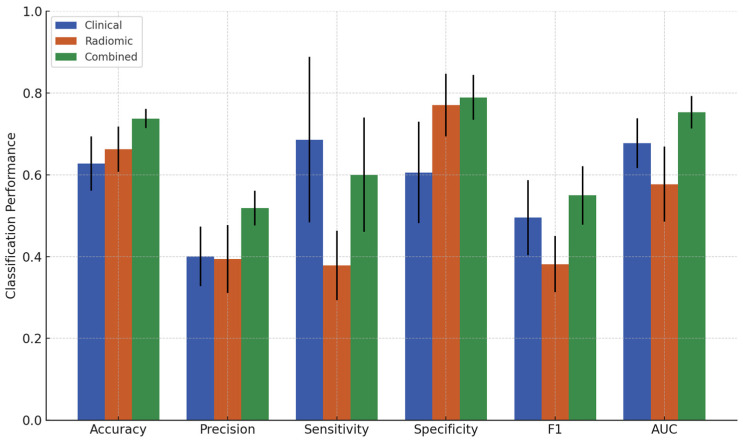
Performance metrics comparison of clinical, radiomic, and combined feature sets for predicting response vs. non-response (criterion 2).

The classification performance of the XGBoost models obtained from the three feature sets for criterion 1 and criterion 2 is summarized in Table 3 and Table 4, respectively. Additionally, bar plots of these results are presented in Figure 3 and Figure 4. The three feature sets are the clinical feature set, the radiomic feature set, and the combined feature set. The classification performance from the clinical feature set served as the baseline for comparison against models incorporating radiomic features. For criterion 1, the clinical feature set yielded an accuracy of 68.2%, precision of 91.4%, sensitivity of 64.2%, specificity of 80.0%, F1-score of 0.739, and an AUC of 0.811. For criterion 2, the clinical feature set achieved an accuracy of 62.7%, precision of 40.1%, sensitivity of 68.6%, specificity of 60.5%, F1-score of 0.495, and AUC of 0.677. The radiomic feature set performed worse than the clinical feature set in all metrics, except for sensitivity (73.9%) for criterion 1 and specificity (77.0%) for criterion 2. However, the combined feature set significantly improved overall classification performance for both response criteria. For criterion 1, the combined set achieved an accuracy of 79.6%, precision of 90.7%, sensitivity of 81.3%, specificity of 74.6%, F1-score of 0.855, and AUC of 0.849. For criterion 2, the combined set achieved an accuracy of 73.7%, precision of 51.8%, sensitivity of 60.0%, specificity of 78.9%, F1-score of 0.550, and AUC of 0.752. Statistically significant differences in these performance metrics among the feature sets are presented in Supplementary Tables S4 and S5.

### 3.3. Features Selected

Feature selection results were further analyzed for the combined feature set, which achieved the best classification performance. It is important to note that different sets of features were selected in each of the 10 data partitions. This process ensures that the model performance is assessed on various subsets of data, enhancing the robustness and generalizability of the predictive model. The list of selected features and their selection frequencies across the 10 iterations are reported in Table 5 and Table 6 for criterion 1 and criterion 2, respectively.

For criterion 1, an average of 14.8 features was used over the 10 iterations. In total, 51 features were selected, with 23 of them being selected only once. Among the 28 features that were selected multiple times, 11 features were selected at least five times, consisting of four clinical features and seven radiomic features. Among the seven frequently selected radiomic features, four were generated from intratumoral segmentations and three from peritumoral segmentations. Finally, the seven radiomic features consisted of two from CE-T1 images, three from T2 images, and two shape features. For criterion 2, an average of 14.1 features was used over the 10 iterations. In total, 58 features were selected, with 18 of them being selected only once. Among the 40 features that were selected multiple times, 12 features were selected at least five times, consisting of five clinical features and seven radiomic features. Among the seven frequently selected radiomic features, two were generated from intratumoral segmentations and five from peritumoral segmentations. Lastly, the seven radiomic features consisted of two from CE-T1 images, three from T2 images, and two shape features.

Most notably, four radiomic features were selected under both criteria: (T2 × Intra) GLCM_ClusterShade, (T2 × Peri) GLCM_ClusterShade, (CE-T1 × Peri) GLDM_SmallDependenceLowGrayLevelEmphasis, and (T2 × Peri) GLSZM_LargeAreaLowGrayLevel-Emphasis. GLCM_ClusterShade measures the asymmetry and irregularity of the spatial intensity distribution in the image. GLDM_SmallDependenceLowGrayLevelEmphasis indicates how frequently small regions with lower intensity values appear in the image. GLSZM_LargeAreaLowGrayLevelEmphasis represents the proportion of large, uniform regions with low intensity values.

**Figure 5:**
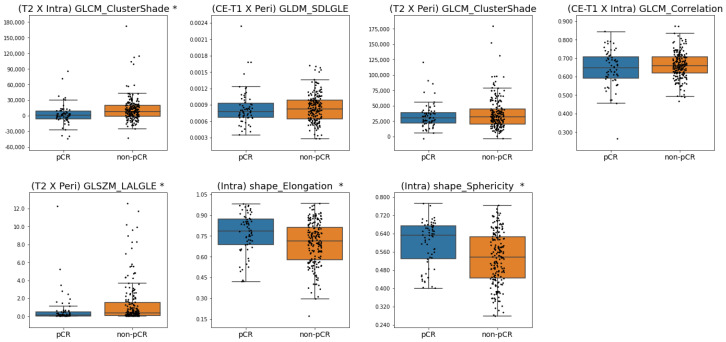
Box plots of the frequently selected features for criterion 1. Statistically significant differences (*p* < 0.05) in distributions between pCR and non-pCR patients are indicated by *. (SDGLE: SmallDependenceLowGrayLevelEmphasis, LALGLE: LargeAreaLowGrayLevelEmphasis).

**Figure 6:**
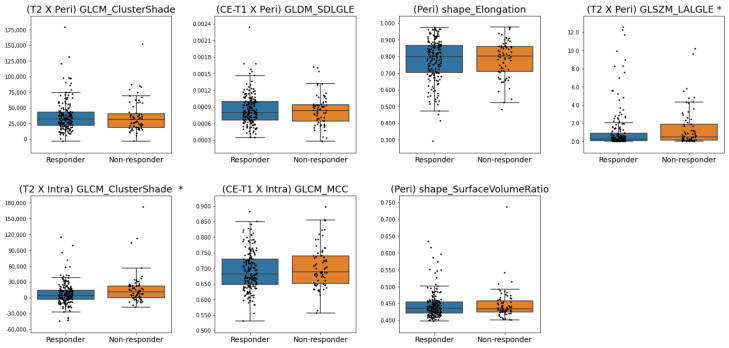
Box plots of the frequently selected features for criterion 2. Statistically significant differences (*p* < 0.05) in feature distributions between responders and non-responders are indicated by *. (SDGLE: SmallDependenceLowGrayLevelEmphasis, LALGLE: LargeAreaLowGrayLevelEmphasis).

Figure 5 and Figure 6 present the box plots of the frequently selected features for criterion 1 and criterion 2, respectively, illustrating differences in feature distributions between the responsive and non-responsive groups. Based on the Mann–Whitney U-test, the distributions of (T2 × Intra) GLCM_ClusterShade, (T2 × Peri) GLSZM_LargeAreaLowGrayLevelEmphasis, (Intra) shape_Elongation, and (Intra) shape_Sphericity were significantly different between pCR and non-pCR patients for criterion 1. For criterion 2, (T2 × Peri) GLSZM_LargeAreaLowGrayLevelEmphasis and (T2 × Intra) GLCM_ClusterShade was significantly different between responders and non-responders. With the two shape features for criterion 1, higher feature values were associated with pCR. In contrast, a higher (T2 × Intra) GLCM_ClusterShade value was associated with pCR and responders for criteria 1 and 2, respectively. In addition, lower (T2 × Peri) GLSZM_LargeAreaLowGrayLevelEmphasis value was associated with the responsive group in both criteria 1 and 2.

**Figure 7:**
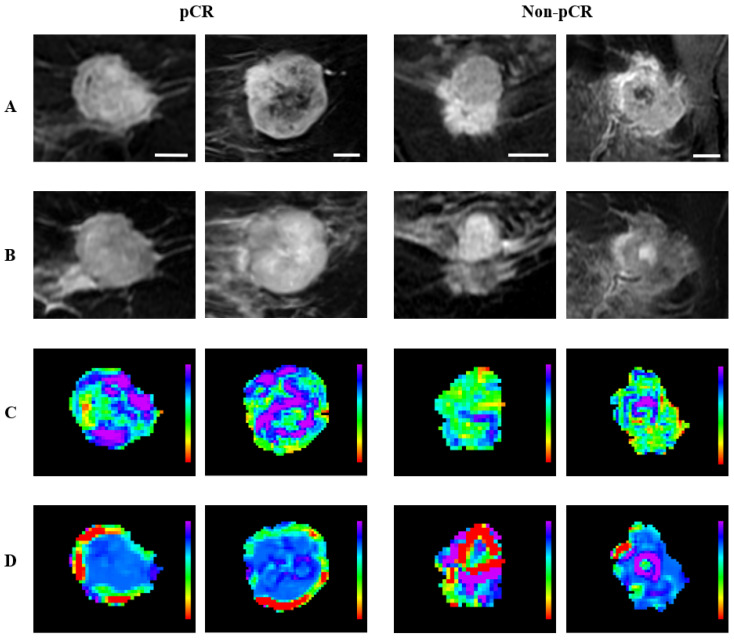
Representative pre-treatment MR images and parametric maps for criterion 1. Each column represents a different tumor corresponding to pCR or non-pCR. (**A**) CE-T1 images; (**B**) T2 images; (**C**) parametric maps of (CE-T1 × Intra) GLCM_Correlation; (**D**) parametric maps of (T2 × Intra) GLCM_ClusterShade. The CE-T1 images include a 10 mm scale bar. Parametric maps are color-coded to represent feature values, with (**C**) ranging from 0 (red) to 0.6 (purple) and (**D**) ranging from −9000 (red) to 3000 (purple).

**Figure 8:**
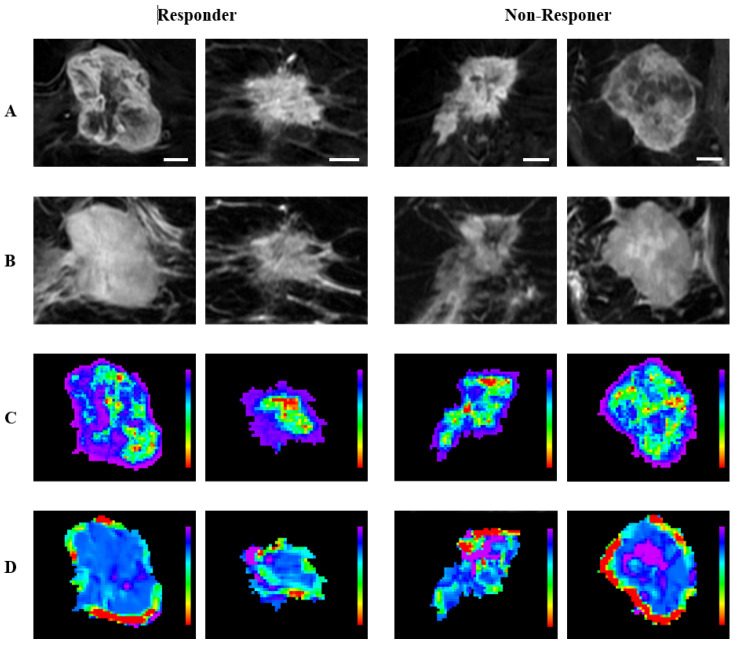
Representative pre-treatment MR images and parametric maps for criterion 2. Each column represents a different tumor corresponding to responder or non-responder. (**A**) CE-T1 images; (**B**) T2 images; (**C**) parametric maps of (CE-T1 × Intra) GLCM_MCC; (**D**) parametric maps of (T2 × Intra) GLCM_ClusterShade. The CE-T1 images include a 10 mm scale bar. Parametric maps are color-coded, with (**C**) ranging from 0.8 (red) to 1 (purple) and (**D**) ranging from −9000 (red) to 3000 (purple).

To highlight the regional distribution of the radiomic features, representative quantitative MRI parametric maps are presented in Figure 7 and Figure 8 for criteria 1 and 2, respectively. For criterion 1, the representative (CE-T1 × Intra) GLCM_Correlation maps of the pCR group exhibit an overall heterogeneous feature distribution with marked feature values in the core region, whereas the non-pCR group showed a more homogeneous feature distribution. For criterion 2, the representative (CE-T1 × Intra) GLCM_MCC maps of responders exhibit larger regions of high feature intensity, especially in the peripheral region, while the maps of non-responders tend to show relatively small peripheral regions of high intensity. (T2 × Intra) GLCM_ClusterShade was the feature selected for both criteria, and the representative feature maps of the responsive groups exhibited a more homogeneous core, whereas the maps of non-responsive groups exhibited a more heterogeneous core with regions of marked feature value. These observations align with the differences in the feature distribution captured by the box plots, where responsive groups and non-responsive groups are associated with lower and higher feature values, respectively.

## 4. Discussion

In this study, XGBoost-based machine learning models were developed using clinical information and radiomic features from pre-treatment MRI to predict breast cancer response to NAC. Radiomic features were determined (“extracted”) from CE-T1 and T2 sequences based on intratumoral and peritumoral segmentations. Two response assessment criteria were examined: one distinguishing pCR from non-pCR, and the other distinguishing responders from non-responders. In criterion 1, pCR was defined as the absence of residual disease in the breast and axillary nodes, while the presence of DCIS was not considered. In criterion 2, response was defined as pCR or partial response with tumor size reduction greater than 30%, while non-response was defined as stable or progressive disease.

Upon statistical analysis of the cohort’s clinical information, histologic grade and receptor status were significantly different between response and non-response groups in both response criteria. Higher histologic grade, ER/PR negativity, and HER2 positivity were associated with better response to NAC. This aligns with clinical knowledge that chemotherapeutic agents such as anthracyclines and taxanes are more effective against aggressive tumors [10]. Histologic grade reflects tumor aggressiveness based on tubule formation, nuclear pleomorphism, and mitotic count. Receptor statuses define the molecular subtypes of breast cancer, which are closely related to tumor biology and NAC response. Luminal A (ER/PR-positive, HER2-negative, low ki-67) and luminal B (ER/PR-positive, HER2-negative or positive, high Ki-67) subtypes tend to respond poorly to NAC, while HER2-enriched (ER/PR-negative, HER2-positive) and triple-negative (ER/PR-negative, HER2-negative) subtypes respond more favorably. The differential response to NAC among breast cancer subtypes can be attributed to both intrinsic tumor biology and the availability of targeted systemic therapies. HER2-enriched and triple-negative breast cancers are generally more aggressive and exhibit higher proliferative activity, which contributes to their increased chemosensitivity. Furthermore, these subtypes benefit from additional therapeutic agents administered during NAC, including targeted therapies for HER2-enriched tumors and immunotherapy for triple-negative disease, both of which have been shown to enhance treatment response. Additionally, significant differences in initial tumor size and nodal status were observed between pCR and non-pCR groups (criterion 1), but not between responders and non-responders (criterion 2). It is postulated that this discrepancy may be attributed to differences in how the two response criteria are defined. In criterion 1, pCR is defined as the complete eradication of the primary tumor and the absence of residual nodal involvement. Therefore, initial tumor size and nodal status are important determinants. In contrast, criterion 2 evaluates response based on relative tumor size reduction without accounting for residual nodal involvement, thereby diminishing the relevance of both initial tumor size and nodal involvement. Lastly, age was the only clinical feature that did not show statistical significance under either response criterion. The lack of statistical significance for these clinical variables is consistent with previous studies. Tran et al. reported that age was not a significant factor in distinguishing pCR from non-pCR [38]. Similarly, Lee et al. found that age, initial tumor size, and nodal status were not statistically significant in differentiating responders from non-responders, where response included both pCR and partial response as defined by RECIST criteria, consistent with the definition used in this study [39].

The classification performance of the radiomic feature set and the combined feature set was compared to that of the clinical feature set, which served as a baseline. This comparison was intended to assess the potential predictive benefit of incorporating MR radiomics with clinical information. For both response criteria, the AUC obtained from the clinical feature set was significantly higher than that of the radiomic feature set. Specifically, the clinical feature set yielded an AUC of 0.811 for criterion 1 and 0.677 for criterion 2, while the radiomic feature set achieved lower AUC values of 0.599 and 0.576 for criteria 1 and 2, respectively. However, combining clinical and radiomic features resulted in statistically significant improvements in AUC, increasing to 0.849 for criterion 1 and 0.752 for criterion 2. The complementary effect of clinical and radiomic features was further demonstrated by examining sensitivity and specificity. For criterion 1, the clinical feature set achieved higher specificity (80.0%) compared to the radiomic feature set (43.1%). Conversely, the radiomic feature set exhibited higher sensitivity (73.9%) than the clinical feature set (64.2%), although this difference was not statistically significant. Since non-pCR was designated as positive and pCR as negative in criterion 1, higher specificity indicates that clinical features play an important role in identifying pCR. Conversely, for criterion 2, the clinical set yielded significantly higher sensitivity (68.6% vs. 37.9%), while the radiomic set achieved higher specificity (77.0% vs. 60.5%). In criterion 2, where non-response was designated as positive and response as negative, the clinical feature set with higher sensitivity was more effective at identifying non-responders, whereas the radiomic feature set with higher specificity was better at identifying responders. When clinical and radiomic features were combined, sensitivity in criterion 1 improved to 81.3%, while specificity in criterion 2 increased to 78.9%, both significantly higher than the results from the clinical feature set alone. In contrast, the difference in specificity in criterion 1 and sensitivity in criterion 2 was not statistically significant between the two feature sets. These results suggest that the clinical features provide a foundation for response prediction, and integrating the radiomic features can further fine-tune the model to enhance overall predictive performance without compromising the baseline performance.

In this study, 10 random partitions of training and test sets were generated. Each training set was used for model building, while the corresponding test set was used for performance evaluation, resulting in 10 independent models. Because feature selection was performed after data partitioning, 10 independent feature sets were generated. This approach was implemented to prevent information leakage and overly optimistic performance estimates. The selected features varied across partitions, and their significance was assessed based on selection frequency. Over the 10 feature sets, 10 and 12 features were selected more than five times for criterion 1 and criterion 2, respectively. The importance of receptor statuses was emphasized again, as all three receptor statuses were selected 10 times for criterion 1. For criterion 2, ER and PR statuses were also selected 10 times, whereas HER2 status was selected 9 times. It should be noted that some clinical features exhibited a discrepancy between statistical significance and selection frequency. For criterion 1, histologic grade and nodal status, which were statistically significant, were selected only three times. Conversely, for criterion 2, age, which was not statistically significant in univariate analysis, was selected 10 times. This discrepancy may arise because statistical significance does not always coincide with predictive significance in machine learning models [40].

Six and seven radiomic features were selected more than five times for criterion 1 and criterion 2, respectively. For criterion 1, the significant radiomic features consisted of two CE-T1 features, three T2 features, and two shape features that are independent of the MRI sequences. For criterion 2, the significant radiomic features included three CE-T1 features, two T2 features, and one shape feature. The CE-T1 sequence captures contrast uptake of the tumor, which reflects tumor vascularity and perfusion [41]. More vascularized tumors, which undergo vascular normalization during treatment, tend to respond better to chemotherapy [42]. The T2 sequence, which is sensitive to water content, may reflect the presence of edema and necrosis [43]. Notably, tumor necrosis is associated with poorer response to NAC, as it may indicate tumor hypoxia, a condition linked to chemoresistance [44]. In terms of segmentation, criterion 1 included three intratumoral and three peritumoral features, while criterion 2 included two intratumoral and five peritumoral features. Intratumoral radiomic features capture information related to tumor heterogeneity [45], while peritumoral radiomic features provide insight into the tumor microenvironment [32]. In addition, peritumoral radiomics has the potential to evaluate the presence of tumor-infiltrating lymphocytes [46], which are associated with better therapy response and prognosis [47]. The utilization of features from both segmentation regions and MRI sequences suggests that a multi-sequential and multi-segmental approach captures multiple pathophysiological tumor characteristics, which meaningfully contribute to response prediction. However, it is important to acknowledge that understanding the precise biological representation of individual radiomic features remains challenging and requires further studies.

A number of studies by other groups have explored the use of MRI radiomics for the prediction of response to NAC in LABC, and these efforts have been reviewed elsewhere [48]. In a study by Braman et al., a model incorporating both intratumoral and peritumoral radiomic features from CE-T1 MRI achieved an AUC of 0.74 for predicting pCR in an independent testing set [32]. Liu et al. developed a multiparametric MRI model incorporating CE-T1, T2, and diffusion-weighted imaging (DWI) from a multicenter dataset, which yielded a maximum AUC of 0.79 for pCR prediction, comparable to the result in this study [26]. The study also indicated that subgrouping data by molecular subtypes could further enhance the predictive performance. Similarly, Granzier et al., in a multicenter study evaluating pCR prediction, reported that clinical models achieved AUCs ranging from 0.71 to 0.77, outperforming radiomic models which yielded AUCs of 0.50 to 0.55, a trend consistent with the findings of this study [27]. However, the combined clinical and radiomic models achieved AUCs of 0.69 to 0.73, which did not result in a statistically significant improvement over clinical features alone. In addition, MRI-based deep learning (DL) methodologies have been investigated for response prediction. In a study by Li et al., a multitask AI system was developed with MRI-based hand-crafted and DL features to classify patients into RCB categories 0–II versus III, and RCB categories 0 and I versus II and III, demonstrating AUCs of 0.94 and 0.92, respectively, in external test sets [49]. The superior performance reported in that study may be attributed to the incorporation of both pre-treatment and mid-treatment MRI. Furthermore, other methodologies evaluated NAC response prediction in LABC using other imaging modalities such as ultrasound and CT [22,23,24,50].

This study could be further improved by implementing auto-segmentation techniques, which would reduce time requirements, minimize operator variability, and ensure feature reproducibility. While image-based harmonization techniques were applied to reduce scanner-related variability prior to feature extraction, feature-based harmonization was not implemented in this study. Future work may incorporate statistical harmonization methods, notably ComBat [51], to adjust radiomic features for scanner-specific effects and further improve the generalizability of predictive models across multi-institutional imaging datasets. Another limitation of this study is the exclusion of patients with breast implants. Although this was necessary to minimize imaging artifacts and variability in feature extraction, it may introduce selection bias, as these patients represent a distinct subgroup not captured by the model. Additionally, this radiomic approach should be validated using a multi-center dataset with a larger cohort. Multi-center datasets introduce variability in image quality due to differences in scanners, acquisition protocols, and operators, which results in a more robust test of the model’s generalizability. Larger datasets could facilitate multi-class analysis, enabling the distinction between non-responders, partial responders, and complete responders, while ensuring an adequate number of training samples for each class. Furthermore, larger and more diverse cohorts would also support subtype-specific modeling. Given that the association between treatment response and prognosis differs across molecular subtypes, future work should consider stratified models tailored to individual subtypes such as Luminal A, Luminal B, HER2-enriched, and triple-negative breast cancers. Finally, while radiomic features can effectively characterize tumor phenotype and heterogeneity, their biological interpretation remains a key challenge. GLCM_Clustershade, for instance, measures the skewness of the spatial intensity distribution, with greater values indicating more pronounced asymmetry in the underlying texture. It is postulated here that this describes the heterogeneity in tumor structure, which is linked to more aggressive tumor behavior. This is similar to histopathological analyses of tumor grade, which subjectively evaluate the disorganization of cell and tissue structure. Future studies incorporating multi-modal data, including histopathological, immunohistochemical, and genomic, transcriptomic, and proteomic information, may provide correlates, improve the interpretability of radiomic biomarkers, and further clarify their association with underlying tumor biology.

## 5. Conclusions

In conclusion, this study demonstrates the potential of predicting breast cancer response to NAC using a machine learning model developed from clinical information and MRI. Incorporating radiomic features from intratumoral and peritumoral segmentations of CE-T1 and T2 images complements the clinical features and significantly enhances the overall classification performance for distinguishing pCR and non-pCR, as well as response and non-response. These prediction models may facilitate more precise and personalized treatment strategies by enabling the early identification of non-responders before treatment initiation.

## Figures and Tables

**Table 1:** Comparison of clinical characteristics between pCR and non-pCR groups (criterion 1).

Characteristics	pCR (*n* = 63)	Non-pCR (*n* = 191)	All (*n* = 254)	*p* Value
Age (year)	50.2 ± 9.0	49.0 ± 11.1	49.3 ± 10.6	0.390
Initial Tumor Size (mm)	36.2 ± 17.4	43.9 ± 24.0	42.0 ± 22.8	0.012
Histologic Grade				0.001
I (%)	1 (1.6%)	12 (6.3%)	13 (5.1%)	
II (%)	17 (27.0%)	92 (48.2%)	109 (42.9%)	
III (%)	45 (71.4%)	87 (45.5%)	132 (52.0%)	
ER				<0.001
Negative (%)	41 (65.1%)	56 (29.3%)	97 (38.1%)	
Positive (%)	22 (34.9%)	135 (70.7%)	157 (61.8%)	
PR				<0.001
Negative (%)	49 (77.8%)	72 (37.7%)	121 (47.6%)	
Positive (%)	14 (22.2%)	119 (62.3%)	133 (52.3%)	
HER2				<0.001
Negative (%)	18 (28.6%)	140 (73.3%)	158 (62.2%)	
Positive (%)	45 (71.4%)	51 (26.7%)	96 (37.8%)	
Nodal Status				0.011
N0 (%)	21 (33.3%)	48 (25.1%)	69 (27.2%)	
N1 (%)	40 (63.5%)	103 (53.9%)	143 (56.3%)	
N2 (%)	1 (1.6%)	31 (16.2%)	32 (12.6%)	
N3 (%)	1 (1.6%)	9 (4.7%)	10 (3.9%)	

**Table 2:** Comparison of clinical characteristics between response and non-response groups (criterion 2).

Characteristics	Response (*n* = 183)	Non-Response (*n* = 71)	All (*n* = 254)	*p* Value
Age (year)	48.5 ± 10.1	51.3 ± 11.8	49.3 ± 10.6	0.075
Initial Tumor Size (mm)	42.9 ± 24.1	39.5 ± 20.0	42.0 ± 22.8	0.561
Histologic Grade				<0.001
I (%)	9 (4.9%)	4 (5.6%)	13 (5.1%)	
II (%)	62 (33.9%)	47 (66.2%)	109 (42.9%)	
III (%)	112 (61.2%)	20 (28.2%)	132 (52.0%)	
ER				<0.001
Negative (%)	86 (47.0%)	11 (15.5%)	97 (38.1%)	
Positive (%)	97 (53.0%)	60 (84.5%)	157 (61.8%)	
PR				<0.001
Negative (%)	103 (56.3%)	18 (25.4%)	121 (47.6%)	
Positive (%)	80 (43.7%)	53 (74.6%)	133 (52.3%)	
HER2				<0.001
Negative (%)	101 (55.2%)	57 (80.3%)	158 (62.2%)	
Positive (%)	82 (44.8%)	14 (19.7%)	96 (37.8%)	
Nodal Status				0.317
N0 (%)	55 (30.1%)	14 (19.7%)	69 (27.2%)	
N1 (%)	101 (55.2%)	42 (59.2%)	143 (56.3%)	
N2 (%)	21 (11.5%)	11 (15.5%)	32 (12.6%)	
N3 (%)	6 (3.3%)	4 (5.6%)	10 (3.9%)	

**Table 3:** Performance metrics of clinical, radiomic, and combined feature sets for predicting pCR vs. non-pCR (criterion 1).

Feature Set	Accuracy (%) ± SD	Precision (%) ± SD	Sensitivity (%) ± SD	Specificity (%) ± SD	F1 ± SD	AUC ± SD
Clinical	68.2 ± 8.8	91.4 ± 4.5	64.2 ± 15.5	80.0 ± 13.4	0.739 ± 0.102	0.811 ± 0.042
Radiomic	66.1 ± 4.6	79.3 ± 3.4	73.9 ± 6.2	43.1 ± 13.0	0.764 ± 0.037	0.599 ± 0.085
Combined	79.6 ± 3.1	90.7 ± 3.6	81.3 ± 6.6	74.6 ± 11.9	0.855 ± 0.028	0.849 ± 0.034

**Table 4:** Performance metrics of clinical, radiomic, and combined feature sets for predicting response vs. non-response (criterion 2).

Feature Set	Accuracy (%) ± SD	Precision (%) ± SD	Sensitivity (%) ± SD	Specificity (%) ± SD	F1 ± SD	AUC ± SD
Clinical	62.7 ± 6.6	40.1 ± 7.3	68.6 ± 20.3	60.5 ± 12.4	0.495 ± 0.092	0.677 ± 0.061
Radiomic	66.3 ± 5.5	39.4 ± 8.3	37.9 ± 8.5	77.0 ± 7.7	0.381 ± 0.069	0.576 ± 0.092
Combined	73.7 ± 2.4	51.8 ± 4.2	60.0 ± 14.0	78.9 ± 5.5	0.550 ± 0.071	0.752 ± 0.040

**Table 5:** Summary of frequently selected features for criterion 1. Features are categorized into clinical (blue), CE-T1 radiomic (green), T2 radiomic (yellow), and shape-based (gray) groups.

Features	#
HER2	10
PR	10
ER	10
Initial Tumor Size	7
(T2 × Intra) GLCM_ClusterShade	6
(CE-T1 × Peri) GLDM_SmallDependenceLowGrayLevelEmphasis	6
(T2 × Peri) GLCM_ClusterShade	6
(CE-T1 × Intra) GLCM_Correlation	5
(T2 × Peri) GLSZM_LargeAreaLowGrayLevelEmphasis	5
(Intra) Shape_Elongation	5
(Intra) Shape_Sphericity	5

**Table 6:** Summary of frequently selected features for criterion 2. Features are categorized into clinical (blue), CE-T1 radiomic (green), T2 radiomic (yellow), and shape-based (gray) groups.

Features	#
ER	10
Histologic Grade	10
Age	10
HER2	9
PR	9
(T2 × Peri) GLCM_ClusterShade	8
(CE-T1 × Peri) GLDM_SmallDependenceLowGrayLevelEmphasis	6
(Peri) Shape_Elongation	6
(T2 × Peri) GLSZM_LargeAreaLowGrayLevelEmphasis	6
(T2 × Intra) GLCM_ClusterShade	5
(CE-T1 × Intra) GLCM_MCC	5
(Peri) Shape_SurfaceVolumeRatio	5

## Data Availability

Data are available upon request (contact the Czarnota Lab at Sunnybrook Health Sciences Centre).

## Supplementary Materials

The following supporting information can be downloaded at: https://www.mdpi.com/article/10.3390/cancers17091520/s1. Table S1. MR scanning parameters for the patients. Values marked with * indicate an approximate average. Table S2. List of features. Table S3. Hyperparameter tuning settings for XGBoost machine learning. Table S4. *p*-values of two-tailed *t*-test comparing classification performance across the three feature sets for criterion 1. Statistical significance with *p* < 0.05 is marked with * and *p* < 0.001 is marked with **. Table S5. *p*-values of two-tailed t-test comparing classification performance across the three feature sets for criterion 1. Statistical significance with *p* < 0.05 is marked with * and *p* < 0.001 is marked with **.
