# Supplementary material for: Pre-Treatment Prediction of Breast Cancer Response to Neoadjuvant Chemotherapy Using Intratumoral and Peritumoral Radiomics from T2-Weighted and Contrast-Enhanced T1-Weighted MRI
Source: Cancers (Basel). 2025 Apr 30;17(9):1520. doi: 10.3390/cancers17091520 (PMC12070997; doi:10.3390/cancers17091520)
Supplement: Supplementary file 1 [file cancers-17-01520-s001.zip › cancers-3550100-supplementary.pdf]

Table S1. MR scanning parameters for the patients. Values marked with * indicate an approximate average.

Scanner	Patients No.	Sequence	Repetition Time (ms)	Echo Time (ms)	Slice Thickness (mm)	Flip Angle	Contrast Agent
GE 1.5T (Signa)	214	T2	2500*	76.14*	3	90°	N/A
		CE-T1	7.3*	3.5*	3	15°	Gadavist
Siemens 1.5T (Aera)	40	T2	6300*	79	3	142°*	N/A
		CE-T1	5.39	2.39	3	15°	Gadavist

Table S2.List of features

Feature Class	Feature Number	Feature Name
**3D Shape**	14	Elongation<br>Flatness<br>Least Axis Length<br>Major Axis Length<br>Maximum 2D Diameter Column<br>Maximum 2D Diameter Row<br>Maximum 2D Diameter Slice<br>Maximum 3D Diameter<br>Mesh Volume<br>Minor Axis Length<br>Sphericity<br>Surface Area<br>Surface Volume Ratio<br>Voxel Volume
**First Order**	18	10 Percentile<br>90 Percentile<br>Energy<br>Entropy<br>Interquartile Range<br>Kurtosis<br>Maximum<br>Mean Absolute Deviation<br>Mean<br>Median<br>Minimum<br>Range<br>Robust Mean Absolute Deviation

		Root Mean Squared<br>Skewness<br>Total Energy<br>Uniformity<br>Variance
**Gray Level Co-occurrence Matrix (GLCM)**	24	Autocorrelation<br>Joint Average<br>Cluster Prominence<br>Cluster Shade<br>Cluster Tendency<br>Contrast<br>Correlation<br>Difference Average<br>Difference Entropy<br>Difference Variance<br>Joint Energy<br>Joint Entropy<br>Informational Measure of Correlation (IMC) 1<br>Informational Measure of Correlation (IMC) 2<br>Inverse Difference Moment (IDM)<br>Inverse Difference Moment Normalized (IDMN)<br>Inverse Difference (ID)<br>Inverse Difference Normalized (IDN)<br>Maximal Correlation Coefficient (MCC)<br>Inverse Variance<br>Maximum Probability<br>Sum Average<br>Sum Entropy<br>Sum Squares
**Gray Level Run Length Matrix (GLRLM)**	16	Gray Level Non Uniformity<br>Gray Level Non Uniformity Normalized<br>Gray Level Variance<br>High Gray Level Run Emphasis<br>Long Run Emphasis<br>Long Run High Gray Level Emphasis<br>Long Run Low Gray Level Emphasis<br>Low Gray Level Run Emphasis<br>Run Entropy<br>Run Length Non Uniformity<br>Run Length Non Uniformity Normalized<br>Run Percentage<br>Run Variance<br>Short Run Emphasis

		Short Run High Gray Level Emphasis<br>Short Run Low Gray Level Emphasis
**Gray Level Size Zone Matrix (GLSZM)**	16	Gray Level Non Uniformity<br>Gray Level Non Uniformity Normalized<br>Gray Level Variance<br>High Gray Level Zone Emphasis<br>Large Area Emphasis<br>Large Area High Gray Level Emphasis<br>Large Area Low Gray Level Emphasis<br>Low Gray Level Zone Emphasis<br>Size Zone Non Uniformity<br>Size Zone Non Uniformity Normalized<br>Small Area Emphasis<br>Small Area High Gray Level Emphasis<br>Small Area Low Gray Level Emphasis<br>Zone Entropy<br>Zone Percentage<br>Zone Variance
**Gray Level Dependence Matrix (GLDM)**	14	Dependence Entropy<br>Dependence Non Uniformity<br>Dependence Non Uniformity Normalized<br>Dependence Variance<br>Gray Level Non Uniformity<br>Gray Level Variance<br>High Gray Level Emphasis<br>Large Dependence Emphasis<br>Large Dependence High Gray Level Emphasis<br>Large Dependence Low Gray Level Emphasis<br>Low Gray Level Emphasis<br>Small Dependence Emphasis<br>Small Dependence High Gray Level Emphasis<br>Small Dependence Low Gray Level Emphasis
**Neighbouring Gray Tone Difference Matrix (NGTDM)**	5	Busyness<br>Coarseness<br>Complexity<br>Contrast<br>Strength

Table S3. Hyperparameter tuning settings for XGBoost machine learning

Hyperparameter	Value
learning_rate	0.01, 0.05, and 0.1

eval_metric	auc
max_depth	3, 5, and 7
min_child_weight	1, 3, 5, and 7
n_estimators	100,250, and 500

Table S4. *p*-values of two-tailed t-test comparing classification performance across the three feature sets for criterion 1. Statistical significance with $p < 0.05$ is marked with * and $p < 0.001$ is marked with **

**Comparison**	**Accuracy**	**Precision**	**Sensitivity**	**Specificity**	**F1**	**AUC**
Clinical / Radiomic	0.388	**	0.175	**	0.680	**
Clinical / Combined	*	0.900	*	0.555	*	**
Radiomic / Combined	**	**	*	**	**	**

Table S5. *p*-values of two-tailed t-test comparing classification performance across the three feature sets for criterion 1. Statistical significance with $p < 0.05$ is marked with * and $p < 0.001$ is marked with **

**Comparison**	**Accuracy**	**Precision**	**Sensitivity**	**Specificity**	**F1**	**AUC**
Clinical / Radiomic	0.150	0.829	**	*	*	*
Clinical / Combined	**	**	0.174	**	*	*
Radiomic / Combined	*	**	*	0.285	**	**
